# Technology-Specific Factors and Online Healthcare Usage Intention Among Older Adults: A Cross-Sectional Study in the Urban Areas of Chongqing, China

**DOI:** 10.3390/healthcare14203477

**Published:** 2026-10-16

**Authors:** Huanchang Liu, Yinuo Zhang, Chengbo Li, Liang Li, Keli Long

**Affiliations:** School of Journalism and Communication, Chongqing University, Chongqing 400044, China

**Keywords:** usage intention, technology-specific factors, older Chinese adults, Chongqing

## Abstract

**Background**: As online healthcare technology aims to support aging, reduce healthcare costs, and offer sustainable solutions, it is important to develop fundamental knowledge on what factors are associated with the intention to use online healthcare services among older persons. Therefore, this study aims to determine how technology-specific factors are associated with older adults’ willingness to use online healthcare services—an aspect that remains underexplored in the available literature. **Methods**: We conducted a cross-sectional survey in 2023 in Chongqing city, Western China, for which 403 urban older adults aged 60 or older were enrolled. **Results**: We found that technology-specific factors—namely, perceived risk (B = −0.100, *p* < 0.001), perceived benefit (B = 0.209, *p* < 0.001), relative advantage (B = 0.261, *p* < 0.001), and compatibility (B = 0.274, *p* < 0.001)—were significantly linked to the intention to use online healthcare services. In contrast, a reported financial strain status of ‘difficult’ (B = −1.024, *p* = 0.019) was negatively related to the intention to use online healthcare services. **Conclusions**: The results of this study identify four technology-specific factors—perceived risk, perceived benefit, relative advantage, and compatibility—which show strong associations with online healthcare usage intention. These results suggest that technology-specific factors may be relevant to older individuals’ perceptions, values, needs, preferences, experiences, and lifestyles, and may be linked to their perceptions of digital health technology. In addition, the results of this study suggest that relative advantage, compatibility, and perceived benefit are positively correlated with online healthcare usage intention, whereas perceived risk is negatively correlated. The findings also demonstrate the applicability of the diffusion of innovations theory and benefit–risk analysis in understanding online healthcare usage intention in older adults. Moreover, these findings and their implications provide specific evidence that the reinforcement of online healthcare usage intention in everyday life depends on technology-related factors, at least in the Chinese context. Better understanding of the theoretical mechanisms underlying the relationships between usage intention and technology-specific factors can aid in the development and implementation of sustainable technology-based solutions for promoting the use of online healthcare services among older adults in Chongqing, China.

## 1. Introduction

Worldwide, two social megatrends that reinforce each other have been identified; namely, the emergence of aging populations and digital technologies, both of which are shaping the future at the local, national, and global levels. Due to sweeping changes in population age structures, there has been an upsurge in demand and expenditure [1] associated with healthcare [2], leading to a situation where the existing supply falls short of demand and health service costs cannot be covered, causing older adults to face problems in terms of access to health services [3]. In this context, particular attention has been paid to technological developments—especially advanced digital health solutions—which may contribute to sustainable improvements in the provision of healthcare services.

The type of digital health technology termed online healthcare is defined as internet-based healthcare services, which has emerged as a complement to traditional care [4] and acts as an extension of offline healthcare services. Online healthcare covers certain connected areas, including third-party health platforms, healthcare software, and mobile health applications, which offer a range of online healthcare services such as online appointment booking, online payment, online medical information retrieval, online consultation/diagnosis/treatment, and online pharmacies, which sell prescription and/or over-the-counter drugs [5,6,7,8]. Several studies have examined digital technologies with the potential to improve the allocation, efficiency, and cost-effectiveness of health services [3], therefore offering low-cost, sustainable solutions for maintaining support among older adults. Hence, it is important to encourage older people to use digital health technologies as they can help to reduce their healthcare costs [9].

With the ongoing development of digital technology, technological tools are altering how individuals engage with their daily tasks. The integration of digital technology and public service programs particularly benefits individuals in accessing medical services online. National investigations have indicated that there were about 411 million users of internet-based healthcare services in China in 2025 [10]; however, online healthcare is not yet ubiquitous, and older adults are a particularly disadvantaged population in this respect due to their lower rates of online healthcare utilization. Hence, it is essential to take urgent measures to enhance the usage intention and rates of online healthcare use in older individuals in China.

Although digital health advancements offer numerous benefits, they also pose difficulties for many individuals [11], especially for those who are older [12]. There is evidence that older adults often experience significant difficulties integrating technological innovations into their daily routines [13]. A previous systematic review has indicated that technology-specific factors were the top barriers to adopting telemedicine worldwide [14]. Technological challenges and changes are the two main sources of resistance to digital shift and engagement [15]—particularly for e-health services [11]—among older adults. In other words, as societies transition toward technology-driven systems, many older individuals encounter barriers to adoption, leading to digital exclusion [12]. Moreover, most previous research has emphasized that the use of modern technologies in the context of everyday activities depends on technological aspects [16,17,18] such as technical compatibility [19], relative advantage [20], the perceived benefits of technological support [21], and perceived risks (i.e., technology-induced concerns or fears) [11]. Therefore, it is important to explore and understand the technology-specific predictors—such as those mentioned above—associated with the intention to use online healthcare, and to establish the paths and mechanisms through which behavioral intentions regarding online healthcare can be successfully improved among older adults.

## 2. Literature Review

### 2.1. Theoretical Mechanisms Underlying the Relationships Between Technology-Specific Factors and Online Healthcare Usage Intention

Rogers identified two main viewpoints in the diffusion of innovations theory [22]; one emphasizes the five key elements of innovation (i.e., relative advantage, compatibility, complexity, observability, and trialability), while the other argues that relative advantage, along with compatibility, influence the rate of technology adoption. By applying these two constructs and the characteristics of innovation, our present study proposes a research model that explains how technology-specific factors are associated with individuals’ intention to use online healthcare technology.

In addition, benefit–risk analysis is a mainstream approach for determining consumption-related behaviors [23]. This model involves two important factors—perceived risk and perceived benefit—which have a strong influence on an individual’s willingness to use new technology [24]. Based on these two factors, our current study employs a research methodology that is designed to better understand the technology-specific predictors of online healthcare usage intention.

Taken together, our current study involves integration of the diffusion of innovations theory and benefit–risk analysis. This integration enables us to more effectively interpret older adults’ diverse feelings and reactions, especially regarding their intention to use online healthcare services and the technology-related reasons for these intentions.

### 2.2. Technology-Specific Factors Associated with Online Healthcare Usage Intention

Previous research has illustrated the critical role of perceived risk in influencing the attitudes and actions of older adults regarding e-health services [11]. Another two previous studies found that perceived risk harms the behavioral intention to use new things [25]. Li et al. discovered that perceived risk negatively impacts the behavioral intention to use remote health management technology [26].

Perceived benefit refers to knowing the concrete benefits of purchasing and/or using an object or new technology [20,27,28]. Past studies have investigated the relationships between perceived benefit and the adoption and use of digital technologies [3,29]; for instance, perceptions of the benefits of online health consultation services positively affect individuals’ intentions to adopt these services [30].

Rogers developed the Diffusion of Innovations Theory, which defines relative advantage as the degree to which a new technology is considered better than its precursor [31,32]. It has been shown that participants prefer to adopt (or had already adopted) technologies that showed better accessibility, safety, and usability (e.g., efficiency, effectiveness, and satisfaction) when compared to conventional devices, methods, and tools [33]. A comprehensive meta-analysis has reported that relative advantage is consistently linked to the adoption of innovative technologies [34]. Previously published studies have noted the impact of relative advantage on the intention to use technology [35]; for instance, the importance of relative advantage has been proven in the context of the consumerization of information technology [36]. Nezamdoust et al. stated that relative advantage had a direct and significant effect on nurses’ use of practical health-related mobile applications [37].

According to Rogers’ Diffusion of Innovations Theory, compatibility—which is second only to relative advantage—is the key innovation element that explains the different rates of adoption among individuals [32,38]. Rogers defined compatibility as “the degree to which an innovation complies with the existing values, past experiences, and needs of potential adopters” [32,39]. It has been indicated that the compatibility of an innovative technology with the technical functionalities of other existing products and with the values, norms, needs, experience, and lifestyles [40] of an individual significantly affects their intention to use the new technology. Compatibility is commonly added to the technology acceptance model to enhance its explanatory power regarding the acceptance of various technologies, including mobile learning [41], Web 2.0 services [42], wearable health monitoring technologies [19], self-driving vehicles [43], online mapping technology [44], and self-service technologies [45]. Compatibility has been identified to significantly and positively influence individuals’ intention to use technology [46]. Similar findings have been shown in previous studies conducted in a number of countries, including China [47,48], Japan and Spain [49], Iran [37], and the Netherlands [50].

### 2.3. Other Factors Associated with Usage Intention

A previous study has reported gender as a predictor of technology adoption behaviors in older individuals [51]. In particular, research has demonstrated that gender is related to the intention to use digital health services, with women reporting a higher intention [52]. Age seems to influence people’s experiences, and could act as a barrier or facilitator for the use of digital health technologies [53]. Yap et al. stated that age is a significant factor associated with technology adoption [51]. Additionally, older people tend to be less willing and have less experience and interest in using e-Health services when compared to younger people [54]. Many studies have indicated that education does have an effect on the use of e-Health in people with chronic diseases [55,56], and those having a higher level of education attainment are more likely to use m-health technology [57]. Socio-economic characteristics may also influence the uptake of digital technology [58]; for example, digital health is generally less accessible for people with lower financial status [59].

Individuals with low socio-economic position have been shown to struggle to adopt e-health programs [60], lag behind in e-health adoption [61], and have more difficulty in accessing m-health services [50]. Economic constraints act as a key barrier to the adoption and effective use of digital health tools [62].

It seems that healthier older people are far more likely to use computers than their unhealthy coevals [63]. Isabelle et al. have confirmed the significant impact of self-reported health status on the intention to use smart wearable systems among older users, where those with better health status are more inclined to adopt such technologies [46]; however, older adults with worse age-related health status are also inclined to adopt smart wearable systems, in order to achieve continuous surveillance of their physical signs [19].

From the literature reviewed above, none of these previous studies have specifically addressed technological aspects that could contribute to improving the usage intention for online healthcare in older adults. This disparity highlights the importance of an in-depth examination of the relationships between technology-specific factors and online healthcare usage intention among older adults in China. In the current study, we present evidence pertaining to these relationships among older people from the urban areas of Chongqing, China.

## 3. Research Method

### 3.1. Data

After receiving study approval from the Ethics Review Board of the School of Journalism and Communication, Chongqing University, a cross-sectional questionnaire with closed-ended questions was used to conduct a survey from October to December in 2023 in urban areas of Chongqing, China. Using a cluster sampling approach, we enrolled 403 urban older adults aged 60 or older who lived in Shapingba district of the Chongqing municipality.

**Figure 1:**
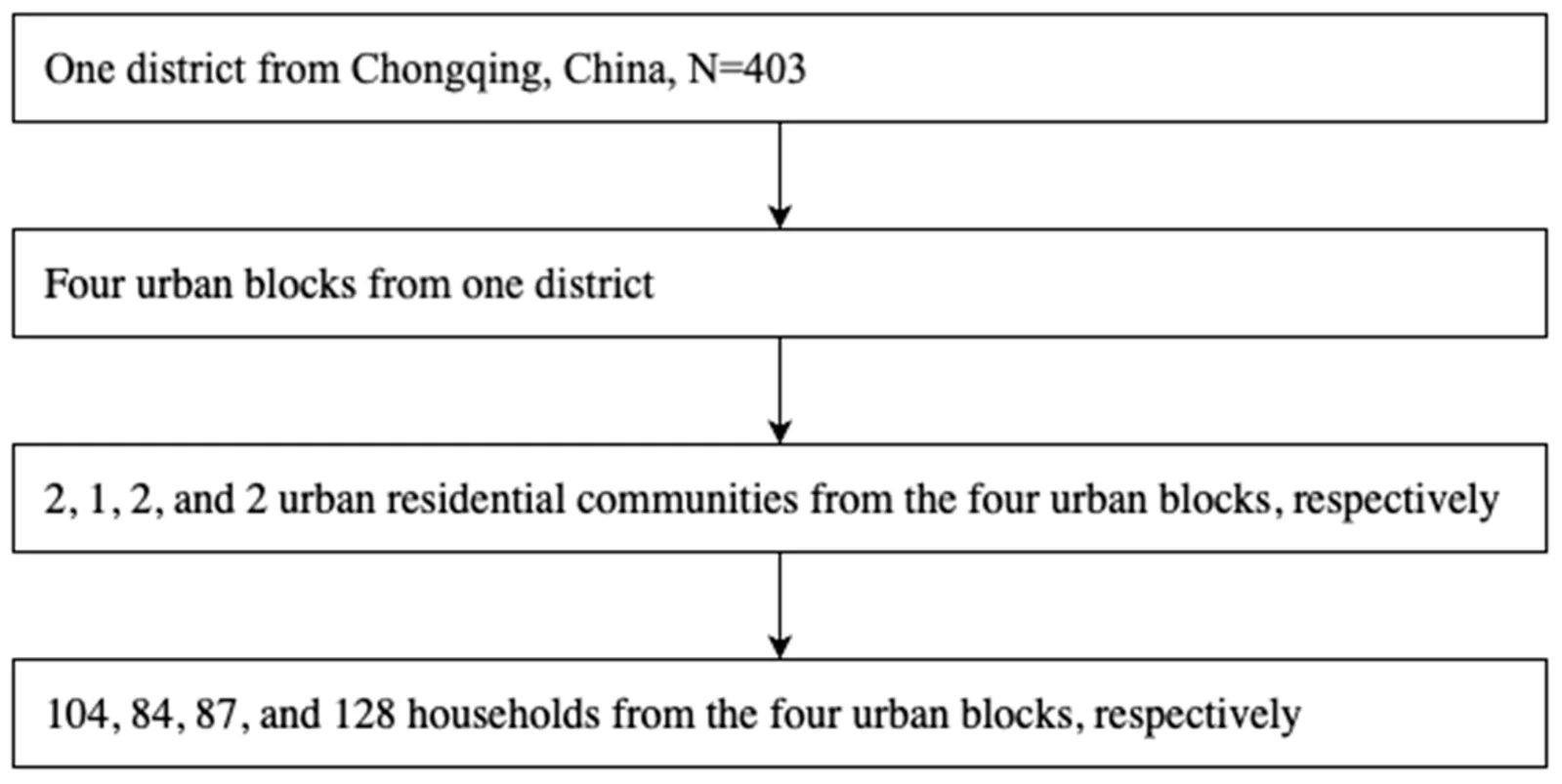
Survey sample selection flowchart.

As described in Figure 1, The process of survey sampling included four stages. In the first stage, we selected one district (Shapingba district) from Chongqing, China. In the second stage, we selected four urban blocks from Shapingba district; namely, Huxi, Zengjiazhen, Shapingba, and Yubeilu. In the third stage, we selected two urban residential communities (Huxi garden and Huama) in Huxi block, one community (Longqiao community) in Zengjiazhen block, two communities (Shazhengjie and Yinshuicun) in Shapingba block, and two communities (Shuangxiangzi and Wusicun) in Yubeilu block. In the fourth stage, households were selected from the selected urban residential communities, resulting in data for 403 urban households, including 104, 84, 87, and 128 households in Huxi block, Zengjiazhen block, Shapingba block, and Yubeilu block, respectively. Within the selected households, in the case of households with more than one person aged 60 or older, one individual was selected at random using the Kish table. Therefore, we enrolled 403 urban older adults aged 60 or older.

In addition, informed written consent was obtained prior to the formal interview. A paper-assisted and in-person structured interview was conducted to promote effective communication with respondents and to ensure successful questionnaire completion. A total of 425 questionnaires were issued and 403 valid questionnaires were returned and collected in our present study, resulting in an effective response rate of 94.82%.

### 3.2. Variables

In our study, the dependent variable was the online healthcare usage intention of individuals, while the independent variables were perceived risk, perceived benefit, relative advantage, and compatibility.

The controlled attributes included demographic variables (gender, age), socio-economic information (educational level, financial strain), and health status (self-rated health, suffering from illness in the past two weeks).

### 3.3. Measurement Instruments

In the questionnaire, the dependent variable (online healthcare usage intention) and the independent variables (perceived risk, perceived benefit, relative advantage and compatibility) were measured using a 5-point Likert scale ranging from 1 (strongly disagree) to 5 (strongly agree). The English scales assessing usage intention, perceived risk, perceived benefit, relative advantage, and compatibility were translated and cross-culturally adapted for Chinese-speaking respondents. Following established guidelines (Beaton et al., 2000), two bilingual researchers independently forward-translated the items into Chinese; a third back-translated them; discrepancies were resolved by consensus. An expert panel assessed semantic, idiomatic, experiential, and conceptual equivalence. Pilot test with five respondents from the target population assessed item clarity and comprehensibility, with minor wording changes; scales were reliable and valid.

#### 3.3.1. The Usage Intention for Online Healthcare

The online healthcare usage intention was measured using a 3-item scale drawn from Mensah et al. [64]. The three specific items were: “I intend to try to use online healthcare in the future,” “I hope to use online healthcare frequently in the future,” and “I would probably recommend online healthcare to people around me.” In the study sample, the Cronbach’s alpha for the 3 items was 0.899. The 3 items were summed to create an overall score with a range of 3–15, with higher scores indicating a stronger online healthcare usage intention.

#### 3.3.2. Technology-Specific Factors

##### Perceived Risk

Based on the work of Peter et al. [65] and Deng [66], perceived risk was assessed using a 5-item scale. The five specific items were as follows: “I am concerned over any financial loss due to the improper use of online healthcare,” “I am concerned for the time lost due to the improper use of online healthcare,” “I am concerned for the physical injury due to the improper use of online healthcare,” “I am concerned for the psychological stress due to the improper use of online healthcare,” and “I am afraid that personal health information would be disclosed or abused due to the improper use of online healthcare.” In our sample, the Cronbach’s alpha for these 5 items was 0.903. An overall score of perceived risk was calculated, resulting in a range of 5–25, with s higher cumulative score indicating a higher level of perceived risk.

##### Perceived Benefit

Gong [30] measured perceived benefit using the six following items: “Using online healthcare can be of benefit to me in managing my health,” “ Using online healthcare can increase my knowledge of my own health conditions,” “Using online healthcare can help to relieve my stresses draw from the symptoms or worries about symptoms,” “Using online healthcare can be contributed to my health,” “Save time by converting going to the hospital to using online healthcare,” and “Save money by converting going to the hospital to using online healthcare.” In our sample, the Cronbach’s alpha for these 6 items was 0.899. The scores from the six items with equal weighting were summed to create an overall score, resulting in a score with a range of 6–30, where a higher cumulative score indicates greater perceived benefit.

##### Relative Advantage

Choudhury [31] measured relative advantage using the following three items: “I would find it more convenient to learn myself about health-related status through online healthcare platforms,” “I would find it more convenient to obtain, collect, manage, understand and use health-related information and service through online healthcare platforms,” and “I would find it more convenient to engage in health-related management for myself through online healthcare platforms.” In our sample, the Cronbach’s alpha for these 3 items was 0.917. The range of the total score was 3–15, computed by adding the scores of each item with equal weighting, where a higher cumulative score indicates greater relative advantage.

##### Compatibility

In the light of Zhao’s research finding [47], compatibility was measured using the following three items: “Using online healthcare is completely compatible with my current health status and demand,” “I think that using online healthcare fits well with my value and philosophy of life,” and “Using online healthcare fits into my experience, habits and the way I live.” In our sample, the Cronbach’s alpha for these 3 items was 0.913. Following the approach for relative advantage detailed above, the scores for compatibility ranged from 3 to 15, where a higher cumulative score indicates better compatibility.

#### 3.3.3. Other Variables

Demographic variables included gender (0 = male, 1 = female) and age. For age, those who were between 60 and 69 years old were coded as 1, those between 70 and 79 years old were coded as 2, and those aged 80 or older were coded as 3.

Socio-economic variables included educational attainment and financial strain. Educational attainment was a 4-response categorical variable (1 = illiterate or primary school, 2 = junior high school, 3 = polytechnic school or senior high school, 4 = college and above). Financial strain was measured via the single question “How do you report your financial strain condition now?” Respondents were asked to rate this item on a five-point Likert scale (1 = more than sufficient, 2 = good sufficient, 3 = approximately sufficient, 4 = somewhat difficult, 5 = very difficult). Due to the severe skewness of the distribution in this study, we collapsed this variable into the three following categories: sufficient (more than sufficient and good sufficient), approximately sufficient, and difficult (somewhat difficult and very difficult).

Health status variables comprised self-rated health and suffering from illness in the past two weeks. For self-rated health, participants were asked a single item: “How do you assess your health situation now?” Their responses were scored on a 5-point Likert scale (1 = very bad, 2 = bad, 3 = fair, 4 = good, 5 = very good). Again, due to the severe skewness of the distribution in the present study, we collapsed this variable into three categories, as follows: bad (very bad and bad), fair, and good (good and very good).

We used a single item to measure suffering from illness in the past two weeks: “Did you suffer from any illness in the past two weeks?” The answer to this item was coded as follows: 0 = no, 1 = yes.

### 3.4. Statistical Analysis

We applied the IBM SPSS 29.0 software (SPSS Inc., Chicago, IL, USA) to calculate descriptive statistics and perform multiple linear regression, in order to determine whether the selected technology-specific factors were associated with the respondents’ online healthcare usage intention. Moreover, there were any missing data, we set this sample’s statistical significance at 0.05. First, descriptive statistics were applied to calculate the frequencies (percentages), means (standard deviations), and F/*t*-test results for all variables. A F/*t*-test was used to compare the means of usage intention according to different characteristics. Second, construct validity was assessed via CFA; convergent validity was assessed using standardized factor loadings, CR, and AVE; and discriminant validity was assessed using the Fornell–Larcker criterion and the HTMT. Third, we conducted the tests and diagnostics to discuss the main assumptions underlying the regression analysis, the linear hypothesis, heteroscedasticity, distribution of residuals, outliers and influential observations, independence of errors were checked. And then a multiple linear regression model was calculated for the present sample, and the regression non-standardized coefficients (B), standardized coefficient (β), confidence intervals (95% CI) and *p*-values, tolerance, and VIF were reported.

Based on the reviewed literature and the research method detailed above, the two equations for the multiple linear regression were as follows:

Model I: y_i_ = β_0_ + β_i_ X_i_ + ε_i_ (_i_ = 1–4)

Model II: y_j_ = γ_0_ + β_j_ X_j_ + ε_j_ (_j_ = 1–10)

y_i_ denotes the dependent variable usage intention in Model I;

y_j_ denotes the dependent variable usage intention in Model II;

X_i_ denotes the four independent variables (i.e., perceived risk, perceived benefit, relative advantage, and compatibility);

X_j_ denotes the 10 variables, including the 4 independent variables mentioned above and the 6 controlled variables (gender, age, educational attainment, financial strain, self-rated health, and the illness condition in the past two weeks);

β_0_ denotes the yi intercept (constant term) in Model I, while γ_0_ refers to the y_j_ intercept (constant term) in Model II;

ε_i_ denotes the error term (also known as the residual) in Model I;

ε_j_ denotes the error term (also known as the residual) in Model II.

## 4. Results

### 4.1. Characteristics of the Sample

As shown in Table 1, the mean usage intention score was 10.33. In addition, the average scores for the four technology-specific factors were 18.19, 21.57, 10.67, and 10.18 for perceived risk, perceived benefit, relative advantage, and compatibility, respectively. Significant differences in usage intention existed in the present sample in terms of the categorical variables age, education attainment, financial strain, self-rated health, and suffering from illness in the past two weeks. We also found significant correlations between usage intention and the three continuous variables perceived benefit, relative advantage, and compatibility.

### 4.2. Construct Validity

For perceived benefit, relative advantage and compatibility, we conducted a confirmatory factor analysis (CFA) to test the construct validity.

The CFA results are presented in Table 2, Table 3, Table 4 and Table 5. The standardized factor loadings of the six items for perceived benefit were 0.815, 0.856, 0.813, 0.875, 0.672, and 0.606, respectively. The standardized factor loadings of the three items for relative advantage were 0.907, 0.859, and 0.893, respectively. The standardized factor loadings of the three items for compatibility were 0.851, 0.940, and 0.861, respectively. The CR values for perceived benefit, relative advantage, and compatibility were 0.900, 0.917, and 0.915, while the AVE values were 0.607, 0.786, and 0.783, respectively, supporting the measures’ convergent validity.

For discriminant validity, perceived benefit showed satisfactory results. In particular, the square root of the average variance extracted (AVE) was 0.781; the correlations with relative advantage and compatibility were 0.742 and 0.687, respectively; and the HTMT values were 0.759 and 0.714. Relative advantage and compatibility were strongly correlated (r = 0.893). Their square root average variance extracted (AVE) values (0.886 and 0.885) were slightly below this correlation, and the HTMT value (0.889) exceeded the 0.850 strict threshold but was below the 0.90 lenient threshold. Therefore, the discriminant validity between relative advantage and compatibility was marginal and not fully supported.

It is particularly important to emphasize that, despite the unsatisfactory discriminant validity between relative advantage and compatibility, they are two key variables in the diffusion of innovations theory, each with its own independent definition, measurement, and theoretical contribution. If either variable were removed or the two were merged in our current study, the integrity of the diffusion of innovations theory—as cited in our research—would be undermined. Accordingly, relative advantage and compatibility are retained as two distinct variables in our current study.

### 4.3. Linear Regression Model

As detailed in Table 6, the R^2^ and adjusted R^2^ values for Model I were 0.555 and 0.550, respectively, while those fore Model II were 0.569 and 0.553, respectively. The R Square Change (ΔR^2^) was 0.015 between Model I and Model II; however, it should be noted that the increase in explained variance was not statistically tested. Although Model II yielded a numerically higher R^2^, the increase in explained variance was not statistically significant. Therefore, the additional predictors did not significantly improve the Model II beyond Model I.

As described in Table 7, two main indices were used to assess the multicollinearity in linear regression model I, which showed an acceptable level of multicollinearity, as indicated by the tolerance (>0.1) and VIF (<5) values. In other words, there was no multicollinearity among all the independent variables.

Furthermore, the regression model presented an R square value ranging up to 0.55, indicating that the variables introduced in this model could explain 55% of the variance in usage intention. Perceived risk (B = −0.100, *p* < 0.001) was significantly and negatively associated with online healthcare usage intention. Meanwhile, perceived benefit (B = 0.218, *p* < 0.001), relative advantage (B = 0.285, *p* < 0.001), and compatibility (B = 0.265, *p* < 0.001) were significantly and positively related to the intention to use online healthcare.

As detailed in Table 8, regression model II was adjusted to include other factors in order to examine whether additional factors—such as demographic attributes, socio-economic status, and health status—were significantly associated with usage intention. All the tolerance (>0.1) and VIF (<5) values suggested that no multicollinearity remained among the independent variables. The regression model presented an R square value ranging up to 0.553, indicating that the variables introduced in this model could explain 55.3% of the variance in usage intention.

Among the participants, perceived risk (B = −0.100, *p* < 0.001) was still negatively linked to the intention to use, while perceived benefit (B = 0.209, *p* < 0.001), relative advantage (B = 0.261, *p* < 0.001), and compatibility (B = 0.274, *p* < 0.001) remained positively related to the intention to use. In addition, those who reported a higher financial strain status (B = −1.024, *p* = 0.019) had less intention to use online healthcare (Table 8). In summary, participants who obtained higher levels of perceived risk and/or reported a financial strain status of “difficult” had lower intention to use, while participants with higher levels of perceived benefit, relative advantage, and compatibility had higher intention to use.

### 4.4. Suppression Effect for Perceived Risk

We conducted a statistical analysis to test the suppression effect for perceived risk.

#### 4.4.1. The Zero-Order Correlation

As can be seen from Table 9, the zero-order correlation between perceived risk and usage intention was weakly positive and non-significant (r = 0.065, *p* = 0.191). However, when including other technology-related factors, the multiple regression models (see Table 7 and Table 8) indicated that the standardized coefficient for perceived risk was significantly negative (Model I: β = −0.149, *p* < 0.001; Model II: β = −0.148, *p* < 0.001). As the β values in Model I and Model II had greater magnitude than the zero-order correlation (r = 0.065), and the direction between the β and r values was opposite, this phenomenon suggested a possible suppression effect.

#### 4.4.2. Hierarchical Regression

In the first stage, linear regression model 1—with perceived risk introduced as an independent variable—was found to explain 0.2% of the variance in usage intention (Table 10). Thus, among the participants, perceived risk (β = 0.065, *p* = 0.191) was not statistically significantly associated with usage intention in this model (Table 11).

In the second stage, regression model 2 (Table 12) was adjusted to include the other technology-specific factors; namely, perceived benefit, relative advantage, and compatibility. The variables used in the regression analysis explained 55.0% of the variance in usage intention for the participants. In this model, perceived risk (β = −0.149, *p* < 0.001) was statistically significantly associated with usage intention.

Based on the results presented in Table 11 and Table 12, we found that the β value of perceived risk was positive but not significant in model 1 (Table 11), while it was negative and significant in model 2 (Table 12); meanwhile, the R square change (ΔR^2^) was significant. Therefore, the finding was consistent with a possible suppression effect.

#### 4.4.3. Formal Test: PROCESS Model 4

As detailed in Table 13, we used the PROCESS macro (Model 4; Hayes, 2022) with perceived risk as the independent variable (X), usage intention as the dependent variable (Y), and perceived benefit as the mediator (M). We used 5000 bootstrap samples and 95% confidence intervals. The sample size was N = 403.

From the results shown in Table 13, we found that the direct effect of perceived risk on usage intention was significantly negative (−0.108), whereas the indirect effect of perceived risk on usage intention was significantly positive (0.152). These two effects had opposite signs, and neither confidence interval included zero. This pattern indicated inconsistent mediation or a suppression effect.

In addition, we confirmed a direct path (namely, c′) indicating that perceived risk was directly and negatively related to usage intention (c′ = −0.108); path a indicated that perceived risk was positively linked to perceived benefit (a = 0.344); and path b indicated that perceived benefit was positively relevant to usage intention (b = 0.443). The indirect path was equal to a × b and, therefore, the indirect effect was positive (a × b = 0.152). Meanwhile, the total effect—namely c = c′ + a × b = −0.108 + 0.152 = 0.044—exactly matched the total effect produced by the model. This explains why the total effect was not significant; that is, because of the positive indirect path and the negative direct path offsetting each other.

Based on the zero-order correlation, the effects of these two paths almost completely offset each other, resulting in a total correlation between perceived risk and usage intention that was near zero and non-significant. Only after controlling for perceived benefit did the net negative effect of perceived risk emerge.

## 5. Discussion

The findings of the present study indicated the associations between the four technology-specific factors (i.e., perceived risk, perceived benefit, relative advantage, and compatibility) and the willingness to use online healthcare among older adults living in urban environments in Chongqing, China. These results suggest that older participants’ diverse reactions as to why they are or are not willing to use online healthcare services can generally be explained based on these four factors, which emerge as important elements linked to older individuals’ perceptions and emotions in response to new technology. Moreover, the results provide specific evidence that reinforcement of the online healthcare usage intentions of older adults in everyday life depends on technology-based factors in the Chinese context.

Perceived risk was a possible negative predictor of online healthcare usage intention; the greater the perceived risk, the more detrimental it was regarding older adults’ willingness to use such technology. This is consistent with previous research; for instance, some studies have found that perceived risk was a significant deterrent regarding the intention to use technology such as mobile ICT for health promotion [67], a mobile chronic disease management system [68], and information services in an online health community [69]. Meanwhile, our results raised an interesting question: Why did perceived risk potentially hinder online healthcare usage intention among older individuals?

In our current research, perceived risk referred to certain types of health risk, financial risk, privacy risk, and psychological risk that arise when an individual uses online healthcare services. Perceived risk thus emerged as a factor that was negatively associated with the usage intention across the four dimensions.

Perceived health risk reflected individuals’ concerns about the wrong diagnosis or delayed treatment. Due to the uncertainties related to healthcare quality, the potential perceived risks of online healthcare far outweighed the benefits of having it. Lack of face-to-face consultations and physical examinations would restrict the accuracy and depth of online communication, which could lead to misdiagnosis or delayed treatment, thereby causing harm to older persons’ health. When older people perceived these health risks, they tended to avoid using online healthcare services.

Perceived financial risk pertained to fears of financial loss or harm from using unfamiliar technologies [11]. An unclear fee or non-transparent price provided through an online healthcare platform would increase older individuals’ concerns about the burdens of treatment, potentially hindering their willingness to use such services. Similar findings have illustrated that the risk of financial loss was an influential factor regarding the adoption of m-health services by older adults in the UK [70].

Perceived privacy risk reflected anxiety about the security of personal health information. If an older person wanted to use an online healthcare service, they would need to satisfy the entrance requirements for the online healthcare platform, which may require registering with their real name and personal information (e.g., gender, age, and past illnesses and chronic conditions), potentially leading to concerns about the security and privacy of their personal health information; put another way, they might live in fear of their personal information being leaked or illegally stolen and used. Due to the age, experience, and roles of older adults in society, they tended to be more conservative and cautious about the privacy risks relating to online healthcare platforms, and their perception of privacy risks might thus correspond to a lower willingness to use online healthcare services.

Perceived psychological risk reflected the psychological burden associated with using unfamiliar technology. The digital divide referred to the subject of the unequal access to and use of new media in the second half of the 1990s, which has brought the important issue of social inequality in information access into both scholarly and political agendas. Prior digital divide-related research has pointed out that the notion of inequality was generally understood to have four distinct meanings. First, it generally referred to inequality in technological opportunities; second, it emphasized inequality in social, cultural, and information capital and resources; third, it denoted the inequality of positions and power in social networks, which could lead to unequal participation in society; and, forth, it has more recently begun to focus on inequalities in skills, capabilities, and interests in the context of the use of digital media in daily life [71]. Furthermore, one of the main aspects of the digital divide was inequality in capabilities or skills [72]. The digital divide has caused older adults to feel confused and anxious when exposed to online healthcare technology, thereby increasing their perceived risk of psychological stress. In particular, a lower capacity to acquire and discern digital information was expected to further amplify perceived risk. Moreover, the pervasive problem of information overload—especially regarding health misinformation—has led older persons to often struggle to distinguish which information is credible. Such perceived risks may be linked to decision-making, thus imposing a further psychological burden for elderly people, which may also be relevant to their reluctance to use online healthcare services.

Rather than treating perceived risk merely as an individual cognitive calculation of technological uncertainty, we reinterpreted it from a relational and socio-material perspective. In this context, perceived risk was shaped by prior experiences with digital technologies and social narratives surrounding the privacy and safety of online healthcare. In particular, participants’ experiences of using—or choosing not to use—digital technologies for health purposes highlighted the vulnerability and uncertainty that people may feel when managing a health problem [73]. Those with extensive experience using health information websites may tend not to trust them due to previous encounters with “questionable” information [74]. Media and privacy experts were quick to sound the alarm about the possibility that health apps could leak sensitive information about people’s health status and geolocation, or that governments could use these data to impose harsh restrictions on freedom of movement through automated decision-making measures that were prone to bias, unfair exclusion, or error [75]. We further hypothesized that these potential relationships between prior experiences or social narratives and perceived risk had a negative influence on usage intention.

A further point that warrants particular discussion was the sign reversal and suppression effect for perceived risk observed in this study. In particular, perceived risk initially appeared to be unrelated to usage intention, as its negative influence was offset by the positive effects of perceived benefit. Perceived risk and perceived benefit were significantly positively correlated, indicating that users’ risk and benefit perceptions of the technology were intertwined. In technology acceptance contexts, users may perceive both greater risk and greater benefit due to deeper cognitive engagement with the technology. Perceived benefit was positive correlates of usage intention, whereas perceived risk was negatively associated with it. Based on the zero-order correlation results, the component of risk perception that covaried with benefit produced a positive indirect effect, masking its true negative effect. This finding suggested that, in technology acceptance models, risk and benefit did not operate independently; rather, competing mechanisms were involved. Therefore, the conventional treatment of the two as independent predictors may need to be revised.

Consistent with previous research conducted in Belgium [76], Saudi Arabia [77], and China [78], the current study illustrated that perceived benefit had a significant positive association with usage intention. This finding implied that older people were more likely to have intent to use online healthcare services when they perceived them to provide obvious benefits. This extended and enriched Rogers’ diffusion of innovations theory, which states that fewer older people will use new technologies if they do not perceive clear benefits [32].

Perceived benefit covered the three aspects of health promotion and quality improvement, access and use of health information, and time-saving. Consideration of these three dimensions enabled a better examination of the relationship between the usage intention and perceived benefit. The perceived benefits of health promotion and quality improvement could be reflected through online healthcare platforms. Adequate resources for health information, such as health tips of sufficient quantity and quality—which could contribute to better health management, and even prevent a potential health crisis—help to establish intention to adopt among older individuals. The perceived benefits of access and use of health information could be leveraged by online healthcare platform. A digital technology provider can meet the need for an online healthcare platform that operates at “any place, any time,” challenging the space and time limitations associated with traditional healthcare. The digital transformation and advancement of healthcare platforms have ushered in a new era of accessibility, making health information and services widely available. This may help to explain the greater online healthcare usage intention observed among older adults. Perceived benefits related to time-saving were readily associated with online healthcare platforms. Based on online healthcare services such as online appointment registration and payment, efficiency and patient experiences can be improved in medical clinics, and the (potentially agonizing) wait times to see a doctor may be reduced. These perceived benefits could be significantly and positively related to online healthcare usage intention.

From a social and relational perspective, perceived benefit should be understood as socially constructed and emerging from relational processes. Older adults rarely engage with online healthcare in isolation; instead, their engagement was embedded in networks comprising family members, peers, clinicians, and institutions. These relationships shaped the extent to which older adults recognized and had confidence in these benefits—namely, in the three key categories of health promotion and quality improvement, access to and use of health information, and time-saving—and whether such perceptions translated into intention to use online healthcare services. Furthermore, the intention to use online healthcare was often shaped by an interdependent set of social and relational factors, including trust in healthcare professionals and institutions, previous experiences with healthcare services, anticipated assistance, existing care practices, and social relationships.

The results of the current study indicated that relative advantage had a significant positive association with online healthcare service usage intention. This association has also been confirmed in other studies [33,37], indicating that online healthcare would be accepted and adopted by older adults only if it was superior to traditional healthcare. In this case, it can be concluded that older adults were willing to adopt online healthcare if a relative advantage of the technology was evident. According to the Diffusion of Innovations Theory [32], the successful diffusion of innovative products depends on widespread acknowledgment of their relative advantages.

By definition, relative advantage referred the degree to which an innovation was perceived as being better than its precursor. Compared to traditional healthcare, online healthcare showed distinct and relative strengths. The conventional process in physical institutions tended to be more intricate, necessitating patients to frequently visit hospitals for follow-up consultations to secure essential prescriptions and medications. Conversely, online healthcare has shifted a portion of the follow-up procedure to digital platforms, thereby streamlining, clarifying, and visualizing the process of seeing a doctor. On the basis of online appointments, payments, and electronic reporting, patients may benefit through “on-the-go” management of healthcare services. As a result, older adults can communicate with their attending physicians regarding disease progression and obtain electronic follow-up prescriptions via video or text communication, all without leaving their homes or navigating through hospitals. This approach alleviated transportation-related stress and minimized certain inconveniences throughout the course of a medical treatment. These positive attributes of online healthcare might have a significant positive association with older adults’ intention to use it.

The finding of the present study also demonstrated the importance of compatibility regarding the willingness to use online healthcare services. Similar evidence has been reported in China [47], the Netherlands [50], and Thailand [37]. Compatibility has been defined as the degree to which technology meets an individual’s needs and complies with both their past experiences and present lifestyle [42]. The diffusion of innovations theory [32] highlights that, when an innovation is compatible with an individual’s current needs and values, it may have a greater likelihood of being adopted by the individual. For older adults, the compatibility of technology with their needs, values, habits, preferences, experiences, and lifestyles has also been posited to have a significant association with online healthcare usage intention; in other words, the higher the degree of compatibility, the more positive association it has with older individuals’ intention to use.

Moreover, online healthcare did not refer to a standalone consumer app. It was embedded in a care system involving patients, clinicians, pharmacists, caregivers, appointments, prescriptions, follow-up, privacy norms, and continuity of care. Therefore, compatibility in the context of online healthcare should not be understood as a purely technological characteristic but, instead, as a relational, contextual, and socio-technical construct. In this regard, compatibility referred to the degree to which digital healthcare become integrated into established practices of seeking, receiving, and managing care, and concerns routines, roles, expectations, trust relations, family involvement, clinical workflows, and the patient–professional relationship. In the context of the current study, Rogers’ diffusion theory already leans toward the relational and contextual perspective, as compatibility includes the fit with an individual’s values, past experiences, and needs. Especially for older adults, usage intention may depend on the compatibility between digital services and their existing healthcare practices, expectations, and relationships with healthcare professionals.

Notwithstanding the theoretical distinction between relative advantage and compatibility and their separate discussion above, the substantial empirical overlap between the two constructs and their inadequate discriminant validity constrained the interpretation of the findings. Although conceptually distinct, the two constructs did not emerge as empirically distinguishable in this sample, suggesting that respondents may have construed them as components of one overarching appraisal of online healthcare. The observed associations between these constructs and usage intention should therefore not be taken as evidence of two independent mechanisms of association.

Apart from the relationships between technology-specific factors and the usage intention, we also confirmed an association with financial difficulty, which entered the model as a controlling covariate. In particular, financial difficulty was inversely associated with online healthcare usage intention. In addition to technological factors, the use of modern technologies in everyday life also depended on economic factors [79]. Due to financial strain, there exist risks, challenges, and inequities in accessing and using digital health services through digital platforms among older adults [80,81]; put another way, those who had a higher level of financial strain were more likely to face barriers to the fulfillment of basic physiological needs, and were less likely to pursue developmental needs and to pay more attention to online healthcare [82]. Therefore, such individuals tended to have less access, resources, and chances to utilize digital health services, which was detrimental to their online healthcare usage intention. Furthermore, the observed relationship between financial difficulty and usage intention highlighted socio-economic inequality and the existence of a social gradient in digital healthcare.

## 6. Implications and Limitations

### 6.1. Implications

Our current findings have significant theoretical contributions. First, the study provides a model for exploring the technology-specific predictors of online healthcare usage intention using the diffusion of innovations theory and benefit–risk analysis. It is important to note that our findings are aligned with aspects of both theories. The results corroborate the theoretical links between usage intention and four technology-specific factors; namely, perceived risk, perceived benefit, relative advantage, and compatibility. These findings provide evidence for the applicability of the diffusion of innovations theory and benefit–risk analysis to the particular situation of older adults in China, and prove their significance in understanding usage intention in the context of digital health.

The present research’s conclusions shed light on detailed characteristics that could ultimately contribute to designing, developing, and implementing online healthcare technology that can meet the needs of the world’s aging populations. First, when designing, developing, evaluating, and implementing new digital health platforms for online healthcare, technology-specific issues such as perceived risk, perceived benefit, relative advantage, and compatibility should be comprehensively considered and addressed, which can be expected to effectively contribute to achieving sustainable digital healthcare ecosystems. Second, to increase the online healthcare usage intention of older adults, the results of this study suggest raising awareness regarding the benefits, risk, compatibility, and relative advantage of such services; in particular, our findings demonstrate the need for interventions that reduce perceptions of risk in online healthcare programs, emphasizing the significance of addressing perceived risk as a major hurdle. In the design stage, safety issues—including health, financial, psychological, and privacy protection—should be comprehensively taken into consideration, based on the significant influence of perceived risk. At the same time, the choice of an online healthcare service is also influenced by its compatibility with older adults’ needs, experience, and lifestyles. Finally, providing financial support and improving individuals’ socio-economic status can boost their intention to use such services.

### 6.2. Limitations

The current study has several limitations. First, for the sampling design (as described in Table 1), our sample comprised a greater proportion of individuals with a higher education level. Meanwhile, although a four-stage cluster sampling procedure was employed, the selection probabilities at each stage were not equal, and we were unable to compute and incorporate sampling weights into the analysis. Furthermore, given the limited number of clusters available for statistical inference, the similarity among individuals within the same block or community may have led to underestimated standard errors, overly narrow confidence intervals, and inflated false positive rates during significance testing. Second, due to the use of cross-sectional data, causal relationships cannot be inferred from the results. Longitudinal research would contribute to addressing these issues, and future studies should conduct follow-up surveys with older persons in order to shed light on the longitudinal effects of relative advantages, compatibility, perceived benefit, and perceived risk on usage intention. Third, although relative advantage and compatibility were retained as separate constructs based on diffusion of innovations theory, the CFA results indicated substantial overlap. The Fornell–Larcker criterion was not fully met for these two constructs and the HTMT value (0.889) exceeded the strict 0.850 threshold, although it was below the 0.900 threshold. These findings suggest that the items may not fully distinguish the two constructs. Future research should refine the questionnaire’s item wording, conduct validation in independent samples, and test whether these constructs can be empirically separated. The present findings should be interpreted with this limitation in mind. Fourth, the relative advantage of online healthcare has been shown during the period of the COVID-19 outbreak in China, as it increases the convenience of online healthcare services for necessity medicines and drugs. However, we failed to consider the effects of the respondents’ past experiences related to the epidemic on their usage intention in the present study. Consequently, future studies should investigate the how the perceived benefits of using online healthcare services are influenced by experiences during previous public health crises. Fifth, critical perspectives on digital health were not considered in depth in this study, and we plan to incorporate further critical perspectives when conducting follow-up studies in the future.

## 7. Conclusions

The results of this study confirm that four technology-specific factors—namely, perceived risk, perceived benefit, relative advantage, and compatibility—have strong associations with older adults’ intention to use online healthcare technology. These associations suggest that technology-based factors may be relevant to older individuals’ perceptions, values, needs, preferences, experiences, and lifestyles, and may be linked to their perceptions of digital health technology. In addition, the results of this study suggest that relative advantage, compatibility, and perceived benefit are positively correlated with online healthcare usage intention, whereas perceived risk shows a negative correlation. The findings also demonstrate the applicability of the diffusion of innovations theory and benefit–risk analysis for understanding online healthcare usage intention in older adults. Moreover, these findings and their implications provide specific evidence that the reinforcement of online healthcare usage intention in everyday life for older adults depends on technology-based factors in the Chinese context. A better understanding of the theoretical mechanisms underlying the relationships between usage intention and technology-specific factors can also aid in developing and implementing sustainable technology-based solutions for the promotion of online healthcare use among older adults in Chongqing, China.

## Figures and Tables

**Table 1:** Characteristics and level of usage intention by different characteristics among the participants (N = 403).

**Categorical Variables**	**N (%)**	**Level of Usage Intention**		
		**Mean (SD)**	***F*** **/** ***t***	***p***
Gender				
Male	195 (48.4)	10.31 (2.24)	−0.2 ^a^	0.842
Female	208 (51.6)	10.36 (2.58)		
Age				
60–69	277 (68.7)	10.59 (2.42)	5.133 ^b^	0.006
70–79	111 (27.5)	9.83 (2.35)		
80+	15 (3.7)	9.40 (2.29)		
Education attainment				
Illiterate or primary school	50 (12.4)	9.46 (2.89)	3.577 ^b^	0.014
Junior high school	87 (21.6)	10.35 (2.11)		
Polytechnic school or senior high school	118 (29.3)	10.21 (2.20)		
College and above	148 (36.7)	10.72 (2.52)		
Financial strain				
Sufficient	87 (21.6)	10.87 (2.39)	12.068 ^b^	<0.001
Approximately sufficient	292 (72.5)	10.35 (2.33)		
Difficult	24 (6.0)	8.21 (2.54)		
Self-rated health				
Bad	108 (26.8)	10.94 (2.21)	5.649 ^b^	0.004
Fair	250 (62.0)	10.18 (2.42)		
Good	45 (11.2)	9.69 (2.62)		
Suffering from illness in the past two weeks				
No	256 (63.5)	10.60 (2.42)	2.932 ^a^	0.004
Yes	147 (36.5)	9.87 (2.35)		
**Continuous Variables**	**N**	**Mean (SD)**	**Correlation with usage intention**	***p***
Usage intention (range: 3–15)	403 (100.0)	10.33 (2.42)	-	-
Perceived risk (range: 5–25)	403 (100.0)	18.19 (3.59)	0.065	0.191
Perceived benefit (range: 6–30)	403 (100.0)	21.57 (3.78)	0.639	<0.001
Relative advantage (range: 3–15)	403 (100.0)	10.67 (2.19)	0.671	<0.001
Compatibility (range: 3–15)	403 (100.0)	10.18 (2.38)	0.666	<0.001

Abbreviation: ^a^ represented for *t* value; ^b^ represented for *F* value.

**Table 2:** Standardized factor loadings for perceived benefit, relative advantage and compatibility.

Item	Perceived Benefit	Relative Advantage	Compatibility
1	0.815	0.907	0.851
2	0.856	0.859	0.940
3	0.813	0.893	0.861
4	0.875	-	-
5	0.672	-	-
6	0.606	-	-

**Table 3:** Composite reliability (CR) and average variance extracted (AVE) for perceived benefit, relative advantage and compatibility.

	CR	AVE
Perceived benefit	0.900	0.607
Relative advantage	0.917	0.786
Compatibility	0.915	0.783

**Table 4:** Fornell–Larcker criterion for perceived benefit, relative advantage and compatibility.

	Perceived Benefit	Relative Advantage	Compatibility
Perceived benefit	0.781	0.742	0.687
Relative advantage	0.742	0.886	0.893
Compatibility	0.687	0.893	0.885

**Table 5:** Heterotrait–monotrait ratio (HTMT) for perceived benefit, relative advantage and compatibility.

	Perceived Benefit	Relative Advantage	Compatibility
Perceived benefit	-	-	-
Relative advantage	0.759	-	-
Compatibility	0.714	0.889	-

**Table 6:** Model Summary ^c^.

Model	R	R Square	Adjusted R Square	Std. Error of the Estimate	Change Statistics		
					R Square Change	F Change	Sig. F Change
I	0.745 ^a^	0.555	0.550	1.622	0.555	123.939	<0.001
II	0.755 ^b^	0.569	0.553	1.618	0.015	1.201	0.284

Note: ^a^. Predictors: (Constant), perceived risk, perceived benefit, relative advantage, compatibility; ^b^. Predictors: (Constant), perceived risk, perceived benefit, relative advantage, compatibility, age group (70–79), age group (80+), gender (female), financial strain (approximately sufficient), financial strain (difficult), education (Junior high school), education (senior high school), education (college and above), self-rated health (fair), self-rated health (good), the illness condition in the past two weeks; ^c^. Dependent Variable: usage intention.

**Table 7:** Linear regression model I regarding technology-specific factors linked to online healthcare usage intention.

Predictors	B (95% CI)	β	*p* Value	Tolerance	VIF
Perceived risk	−0.100 (−0.147,−0.053)	−0.149	<0.001	0.887	1.127
Perceived benefit	0.218 (0.157,0.280)	0.341	<0.001	0.470	2.127
Relative advantage	0.285 (0.151,0.419)	0.258	<0.001	0.293	3.410
Compatibility	0.265 (0.148,0.383)	0.261	<0.001	0.324	3.087
Constant	1.713 (0.626,2.800)		0.002		
Adjusted R Square	0.55				
*F*	123.939				
*p*	<0.001				

Note: CI, confidence interval; B, non-standardized coefficient; β, standardized co-efficient; F represented for F value; *p* represented for *p* value.

**Table 8:** Linear regression model II regarding technology-specific factors linked to online healthcare usage intention (controlling demographic variables, socio-economic status and health status).

Predictors	B (95% CI)	β	*p* Value	Tolerance	VIF
Perceived risk	−0.100 (−0.148, −0.051)	−0.148	<0.001	0.837	1.194
Perceived benefit	0.209 (0.147, 0.272)	0.327	<0.001	0.450	2.224
Relative advantage	0.261 (0.124, 0.399)	0.237	<0.001	0.279	3.589
Compatibility	0.274 (0.154, 0.394)	0.270	<0.001	0.308	3.242
Gender					
Male (reference)					
Female	−0.031 (−0.356, 0.293)	−0.006	0.850	0.954	1.048
Age					
60–69 (reference)					
70–79	−0.339 (−0.712, 0.034)	−0.063	0.075	0.904	1.106
80+	−0.183 (−1.065, 0.700)	−0.014	0.684	0.900	1.111
Education level					
Illiterate or primary school (reference)					
Junior high school	0.401 (−0.175, 0.976)	0.068	0.172	0.447	2.235
Polytechnic school or senior high school	−0.089 (−0.650, 0.472)	−0.017	0.755	0.385	2.594
College and above	0.015 (−0.545, 0.576)	0.003	0.957	0.344	2.910
Financial strain					
Sufficient (reference)					
Approximately sufficient	−0.193 (−0.598, 0.213)	−0.036	0.351	0.764	1.308
Difficult	−1.024 (−1.879, −0.170)	−0.100	0.019	0.614	1.630
Self-rated health					
Bad (reference)					
Fair	−0.283 (−0.853, 0.286)	−0.057	0.328	0.329	3.042
Good	−0.105 (−0.748, 0.537)	−0.019	0.747	0.310	3.223
The illness condition in the past two weeks					
No (reference)					
Yes	0.071 (−0.278, 0.421)	0.014	0.689	0.887	1.128
Constant	2.473 (1.056, 3.889)		<0.001		-
Adjusted R Square	0.553				
*F*	34.116				
*p*	<0.001				

**Table 9:** Correlations among key study variables.

Continuous Variables	1	*2*	*3*	*4*	*5*
1. Usage intention	-	0.065	0.639 ***	0.671 ***	0.666 ***
2. Perceived risk		-	0.327 ***	0.226 ***	0.170 ***
3. Perceived benefit			-	0.689 ***	0.647 ***
4. Relative advantage				-	0.812 ***
5. Compatibility					-

Note: *** *p* < 0.001.

**Table 10:** Models Summary ^c^.

Model	R	R Square	Adjusted R Square	Std. Error of the Estimate	Change Statistics		
					R Square Change	F Change	Sig. F Change
1	0.065 ^a^	0.004	0.002	2.41639	0.004	1.716	0.191
2	0.745 ^b^	0.555	0.550	1.62203	0.550	163.983	<0.001

Note: ^a^. Predictors: (Constant), perceived risk; ^b^. Predictors: (Constant), perceived risk, perceived benefit, relative advantage, compatibility; ^c^. Dependent Variable: usage intention.

**Table 11:** Linear regression model 1 regarding perceived risk linked to online healthcare usage intention.

Predictors	B (95% CI)	β	*p* Value	Tolerance	VIF
Perceived risk	−0.044 (−0.022, 0.110)	0.065	0.191	1.000	1.000
Constant	9.533 (8.310, 10.756)	-	<0.001	-	-
Adjusted R Square	0.002				
*F*	1.716				
*p*	0.191				

**Table 12:** Linear regression model 2 regarding perceived risk, perceived benefit, relative advantage and compatibility linked to online healthcare usage intention.

Predictors	B (95% CI)	β	*p* Value	Tolerance	VIF
Perceived risk	−0.100 (−0.147, −0.053)	−0.149	<0.001	0.887	1.127
Perceived benefit	0.218 (0.157, 0.280)	0.341	<0.001	0.470	2.127
Relative advantage	0.285 (0.151, 0.419)	0.258	<0.001	0.293	3.410
Compatibility	0.265 (0.148, 0.383)	0.261	<0.001	0.324	3.087
Constant	1.713 (0.626, 2.800)		0.002		
Adjusted R Square	0.55				
*F*	123.939				
*p*	<0.001				

**Table 13:** Key effects among key study variables.

Effect	Coeff.	SE	t/BootSE	*p*/95% BootCI
Total effect of perceived risk on usage intention	0.044	0.034	t = 1.310	*p* = 0.191, 95% CI [−0.022, 0.110]
Direct effect of perceived risk on usage intention	−0.108	0.027	t = −4.032	*p* = 0.0001, 95% CI [−0.161, −0.056]
Indirect effect of perceived risk on usage intention	0.152	-	BootSE = 0.033	95% BootCI [0.087, 0.218]
Perceived risk→ Perceived benefit	0.344	0.050	t = 6.934	*p* < 0.0001
Perceived benefit→ Usage intention	0.443	0.026	t = 17.320	*p* < 0.0001

Note: coeff. represented for coefficient; SE represented for standard error; t represented for t-value; BootSE represented for bootstrap standard error; *p* represented for *p*-value; BootCI represented for bootstrap confidence interval.

## Data Availability

The data presented in this study are available upon request from the corresponding author. The data are not publicly available due to privacy restrictions.
